# Supplementary material for: Did you see it? A Python tool for psychophysical assessment of the human blind spot
Source: PLoS One. 2021 Nov 4;16(11):e0254195. doi: 10.1371/journal.pone.0254195 (PMC8568268; doi:10.1371/journal.pone.0254195)
Supplement: Attachment — Submitted filename: BS_Response_to_reviewers_revise.docx [file pone.0254195.s001.docx]

**Responses to reviewers: PONE-S-21-25541**

We are grateful to both reviewers for their constructive comments and positive appraisal of our work. Below we provide point-point responses (in blue) and edits to the revised manuscript are highlighted in red.

**Reviewer #1:**

This paper provides a useful tool for standardising plotting of the blind spot. It is clearly and thoughtfully presented and easy to read. I do not have any major concerns about the paper.

**Response:** Thank you for the assessment of our work.

Minor comments:

I have a couple of minor concerns. The first refers to the window of tolerance used by the eye-tracker. If 1.5 deg tolerance was permitted, then the eye could move across a 3x3 deg region during data collection. That is almost half the width or height of the blind spot. I wish a more realistic window had been chosen. I would have liked to see data with a tolerance of 0.5 deg for the eye-tracker. I suspect that this will not make much of difference with regard to the measured size of the blind spot.

**Response:** We chose 1.5° to smooth our study progress, because the task is demanding and extensive. Before the formal study, we tested a 1° tolerance and we found that fixation failures occurred frequently, even when participants were certain they were fixating, which was frustrating and exhausting, and lengthened testing time.

Examination of eye traces indicates that fixation errors were exacerbated by slight drift of the eye-tracker signal, so that accurate fixations were being classed as inaccurate. This meant that the participant could get broken fixation feedback even when fixating accurately, which explains the sense of frustration. The only way to avoid this would be to include online drift correction, and have regular drift correction steps, which would be laborious and confusing for the participant, and risk the introduction of more serious errors. The 1.5° tolerance threshold was therefore chosen intentionally as the default setting, as a compromise between accuracy and comfort. Each session was done within one hour, and it would be too long if we used a smaller tolerance.

We want to emphasize that the tolerance level is not a fixed parameter, so users can adjust this to suit their needs and/or participant group. Nonetheless, as supported by blind spot estimates and time cost, the default setting of 1.5° appeared to be satisfactory (blind spot estimates were validated by the validation step and they were close to earlier studies).

Note that this tolerance, although it may seem high, is not high with respect to comparable literature that have used eye-tracking during blind spot estimation. For instance, Ehinger et al. (2015, 2017) used 2.6° tolerance.

We now discuss and justify our default tolerance level in the revised manuscript on page 12, lines 184-192, and we provide further context in the responses below.

However, it would have been a bit more reassuring, because if the eye is moving by as much as 3 deg, then the blind spot edge is moving by as much as 3 deg. The reason this does not show in the data is probably because, even though the eyes were permitted to move over a big window, the observers were sufficiently trained that they did not actually move their eyes. But if this tool were to be used widely and users were tempted to use this study as a basis for using a 1.5 deg window, there would occasionally be subjects (particularly when testing patients with visual disability) who would make large eye-movements during testing.

It would be helpful if some data were presented with a smaller tolerance during eye-tracking.

**Response:** We are unable due to the current COVID-19 situation to acquire new data with a smaller tolerance. For the reviewer’s benefit, we have analyzed the eye position data for all blocks and across all observers in our existing dataset to illustrate why the explain why the chosen tolerance window was not detrimental to our blind spot estimates.

Fixation position data are summarised below and plotted in Figs S1, S2, and S3. Fig S1 shows that within-tolerance fixation offsets (fixation position – fixation object) were generally much lower than 1.5°. We analysed the median fixation offsets at the moment that the stimulus was presented or the observer responded, when the fixation position was within the tolerance window. Across all participants, these fixation offsets fell within the range of 0.37° and 1.15°, well below the maximum tolerance of 1.5°. Further breakdown of these data shows that most offsets were <1° (11 blocks < 0.5°; 27 blocks < 0.75°; 24 blocks <1°; 8 blocks >1°). Very similar medians were observed during the border point detection (Fig S2. range = 0.3° and 1.2°; 14 blocks <0.5°; 30 blocks <0.75°; 18 blocks <1°; 8 blocks >1°). So, to the extent that observers may have moved their eyes, it was overwhelmingly within a smaller window than 1.5°.

However, it is almost certain that the above over-estimates the true fixation offset, because the recorded values will include any eyetracker drift. To illustrate this point, we separated the fixation position data into horizontal and vertical components, for each observer across all blocks. We present the data for Observer No. 9 in Fig S3 as an example. In this figure, we included all fixation positions regardless of within-tolerance or not. In both horizontal and vertical dimensions, fixation positions were quite consistent from trial-to-trial (as suggested by the narrow and dark band), but nonetheless drifted over time over the course of some blocks.

This pattern is much more interpretable as a drift of the eye-tracker signal than of the eye position itself. It would be very difficult to fixate systematically away from the centre of the fixation point, and one would not expect this to follow a drift pattern like that observed (or to go in different directions in different blocks). As noted, we did not turn on online drift correction, but corrected drift only at the beginning of each block, in order to make the task simpler and smoother for the participant.

If users want to set a stricter tolerance than the default 1.5°, we would therefore recommend that they should turn on online drift correction, and perform drift correction whenever fixation becomes unstable during a block. We now provide this advice in the manual (3.3 Experiment configuration files, and 6.7 Drift correction), and refer to it in the paper (page 12, lines 184-192). This is an advanced-user feature, and not necessary or appropriate for the main purpose of the tool, which is to define a safe zone for stimulus presentation in blind-spot experiments.

We think that consideration of this issue makes the paper and especially the manual more comprehensive, and we are grateful to the reviewer for raising it. We hope that our analysis and responses provide sufficient reassurance that our tolerance window was appropriate.

Additionally I think a few simulations would make the results interesting. Imagine a 7x6 deg oval blind spot - what would the measured size of the blind spot be if the eye was randomly positioned within a 0.5x0.5 deg window, 1.0x1.0 deg window, 1.5x1.5 deg window on each trial? I think this is an easy simulation to do. It would help us predict the consequences of poor fixation on mapped blind spot size.

**Response:** We did not simulate the data as suggested because (as shown in Fig 3), the eye movements were not randomly distributed within the tolerance window, but instead concentrated at a specific level, as suggested by the dark band, albeit with one drift in the signal. The observed drift and our recommendation of using online drift correction if choosing a smaller tolerance window, are now incorporated in the manual, and mentioned in the paper, as discussed above.

The other concern I have has to do with potential mismatches between some numbers in the text part of the paper and those in the tables. It is possible I might have misunderstood something. "On average (bottom row of Table 2), the right blind spot was located at 16.00° temporal (right) to the fixation (SD = 0.52°, Range = [15.17°, 16.73°], n = 12), and slightly lower than the horizontal meridian (mean = - 2.05°, SD = 1.21°, Range = [-4.33°, -0.50°], n = 12)."
0

I can see the mean values match what I see in Table 2, but the SD and range do not seem to match. Perhaps I missed something?

**Response:** We apologize for the confusion regarding data mismatches between the text and the table. This is because they refer to different measurements of SD, and we had not made this sufficiently clear. For the last paragraph on page 21, the in-text SDs of horizontal and vertical locations are for the 12 means (i.e., **SD(mean(p1), mean(p2), ..., mean(p13))**), while the in-table SDs are the average of 12 individual SDs; the in-text Ranges are the max and min values of 12 individual means (i.e., the **max and min of the "Location (H, V)" column excluding the row "Average"**, of the table), while the in-table Ranges are the **max and min of all 70 estimates** (they are also the Min and Max of column "Location H Range" excluding the last row and that of column "Location V Range" excluding the last row).

We are grateful to the reviewer for pointing this out. It also caused us to reconsider our formula for average SD in the table, which we realise should have been calculated as the square root of the mean variances; similarly, the average standard distance should have been calculated as the square root of the average squared standard distance.

These changes have been made to Table 2, and we have added an explanatory note to the table legend to clarify the calculation of average ranges, SDs and standard distances, and the differences from the SD and range of mean locations reported in text.

"Overall, the blind spot width was 6.28° (SD = 0.62°, CV = 0.10, Range = [5.39°, 7.44°], n = 12), and the height was 7.02° (SD = 0.77°, CV = 0.11, Range = [5.79°, 8.14°], n = 12)." Again, the mean values match Table 2, but again the SD and range do not seem to match.

**Response:** This reflects a similar difference in the nature of the measurements as above, now clarified in Table 2 legend.

**Reviewer #2:**

The manuscript by Ling, Silson, & McIntosh introduces a new software package to psychophysically measure the human retinal blind spot. This is a welcome addition to publicly available tools, since many studies need to measure the location of the retinal blind spot. In the past, many authors have implemented their own tools, but a publicly available tool will surely help to make research on visual scotomata and perceptual filling-in more accessible and more reproducible. In the paper, the authors present the workflow of the software and the various options that were implemented to measure, correct, and verify blind spot boundaries. They go on and validate the tool in detailed measurements of a single subject’s blind spot, as well as with less detailed (yet more realistic) measurement parameters in a group of several subjects. This serves to show the usability and validity of the tool.

I want to commend the authors for providing such a valuable tool, and for presenting this useful assessment of its validity!

I would only ask the authors to address a few minor comments, which would help to clarify some issues.

**Response:** We thank the reviewer for the positive comments. We too hope that this will provide a useful and accessible tool for the vision community.

Figure 1B Caption:
“Note that in the 1st-horizontal-test of “Medium” mode, the separation between the middle and upper paths is the same as the middle-lower separation, and they are predetermined as 1.875°(7.5°/4, or 240 pix/4 when the viewing distance is 57 cm), which is much smaller than a half of a typical blind spot height. The separation between the 1st-vertical-test paths is not predetermined but is set to 1/4 of the estimated blind spot width obtained by the 1st-horizontal-test. The 2nd- horizontal-test has only one path. “

I failed to parse this text on first pass. I guess I don’t understand the placing of the horizontal test paths, and which order they are being run in. It would help to label these in the figure. A more logical procedure would be, after the bs-center is determined after the 2nd horizontal path, to have paths passing through the center of the BS, with additional test points placed equidistantly along the border. Maybe this is something to consider for a future release of the software.

**Response:** On re-reading, we realise that the figure legend was hard to understand. We have re-written the legend for clarity. We now make it more clear that the numbering of the test-paths indicates the order in which they are run. We also make it clear which paths the blind spot location and extent (height, width) are estimated from, and that additional paths provide additional border points to inform about blind spot shape.

We also highlighted in our paper (legend of Fig 1) that full descriptions about "Medium" and "High" are provided in the online manual ("Mode" item of Section 3.3).

The reviewer’s suggestion seems to be that, having determined the blind spot centre, additional border points could be collected by having radial paths passing through the blind spot, with regular angular spacing. This is a good idea, and it is something that we will consider for future developments of the tool.

Page 17: The calculation of trial numbers is a little confusing. Maybe specify in further detail that the sum includes trial numbers for each phase of the experiment.

**Response:** We have clarified the trial numbers in the revised manuscript (page 23 lines 380-382, page 24 lines 384, 385, 387, and 388.).

Experiment 2: It would help to specify from the outset that multiple blocks are run for each observer to be able to assess with-in subject consistency.

**Response:** We agree with the reviewer and have made this clear in the revision (page 25, lines 409-410).

Caption of Figure 3: Maybe don’t label answers as Wrong and Correct, since technically they aren’t either.

**Response:** This point is well taken. We have replaced "Wrong" and "Correct" with "Negative" and "Positive".

Table 1: Wang 2017 TVS?? or MVS

**Response:** Thanks for spotting this typo. It has been corrected to MVS.

**S1 Fig. Effective fixation positions relative to the fixation object throughout the whole testing block for all blocks and observers**. Larger offsets suggest larger distances from the fixation positions to the fixation object, while 0° suggests fixation at the fixation object. Each violin plot incorporates eye position data from all three sections* (Border points detection, Staircase, and Validation). Each violin plot is cut off at the max and min values of the observed data. The width of violin plot is scaled by the data count. The mini box plot inside a violin plot indicates the interquartile range (25 and 75 percentiles) and the median.

*** For the Border points detection section, data are included when the fixation position was within the tolerance (1.5°) and the observer clicked the mouse. For the Staircase and Validation sections, data are included when the fixation position was within the tolerance and the target object was being displayed, so these data were not necessarily effective because they might be excluded from estimation.**

**S2 Fig. Effective fixation positions relative to the fixation object during the Border points detection section* for all blocks and observers**. Larger offsets suggest larger distances from the fixation positions to the fixation object, while 0° suggests fixation at the fixation object. Each violin plot is cut off at the max and min values of the observed data. The width of violin plot is scaled by the data count. The mini box plot inside a violin plot indicates the interquartile range (25 and 75 percentiles) and the median.

*** Data are included when the fixation position was within the tolerance (1.5°) and the observer clicked the mouse.**

**S3 Fig. Fixation positions relative to the fixation object for Observer No. 9 across all blocks**. All fixation positions are included but data beyond 2° are outside the subplots.
